# Partial molar pregnancy in the cesarean scar: A case report and literature review

**DOI:** 10.1097/MD.0000000000011312

**Published:** 2018-06-29

**Authors:** Chen Ling, Jitong Zhao, Xianrong Qi

**Affiliations:** Department of Gynecology and Obstetrics, Key Laboratory of Obstetrics and Gynecologic and Pediatric Diseases and Birth Defects of Ministry of Education, West China Second Hospital, Sichuan University, Chengdu, Sichuan, China.

**Keywords:** case report, cesarean scar pregnancy, histologic examination, literature review, molar pregnancy

## Abstract

**Rationale:**

The incidence of molar pregnancy in the cesarean scar is exceedingly low, however, the disease may carry a high risk of uncontrolled hemorrhage or uterine rupture. So far managements of this disease were rarely reported in literature.

**Patient concerns:**

We reported a 28-year-old woman presented to our hospital with a complaint of amenorrhea for 48 days and vaginal bleeding for 3 days.

**Diagnosis:**

Transvaginal ultrasonography, serum hCG and pelvic MRI confirmed the cesarean scar pregnancy.

**Interventions:**

The patient underwent bilateral uterine arterial embolization and suction evacuation.

**Outcomes:**

The postoperative histologic examination of the tissue revealed a partial hydatidiform mole.

**Lessons:**

Molar pregnancy in the cesarean scar is tough to differentiate from normal cesarean scar pregnancy with serum hCG, sonogram or MRI. This case suggested us that it was necessary to perform a histological examination of postoperative specimen for cesarean scar pregnancy.

## 1 Introduction

Cesarean scar pregnancy (CSP) is a rare type of ectopic pregnancy, with a frequency of 1:1800 to 1:2216 pregnancies, which may lead to uncontrollable hemorrhage.^[1]^ Hydatidiform mole is a subtype of gestational trophoblastic diseases, with the incidence estimated at 1%.^[2]^ Thus, molar CSP is exceedingly rare and may have a high risk of uncontrolled hemorrhage or uterine rupture. Patients with this condition often need blood transfusion, leading to hysterectomy. However, because of the extremely low incidence, the literature shows lack of evidence on the diagnosis and optimal management of cesarean scar molar pregnancy.

Here, we report a case of and review the literature on partial molar CSP, providing experience for further studies.

## 2 Case report

**Figure 1:**
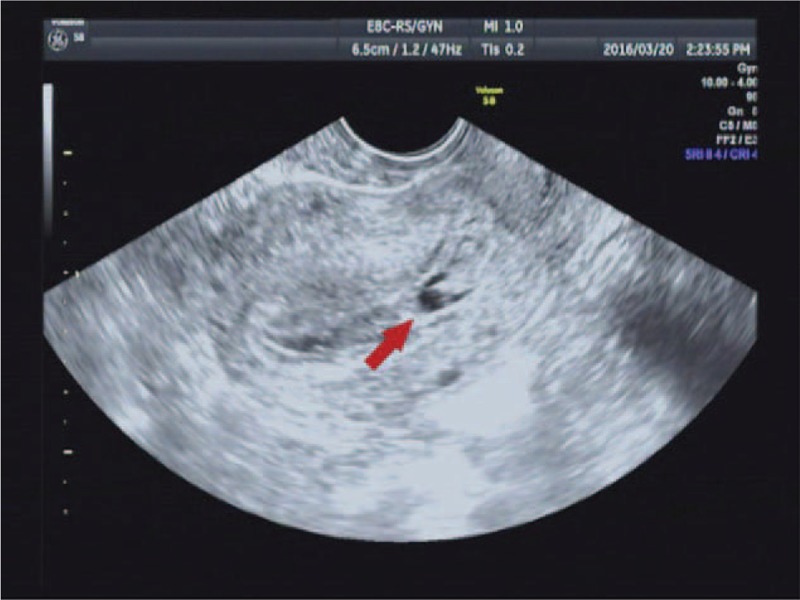
Transvaginal sonogram showing a gestational sac implanted near the previous cesarean scar in the anterior wall of the uterine corpus.

**Figure 2:**
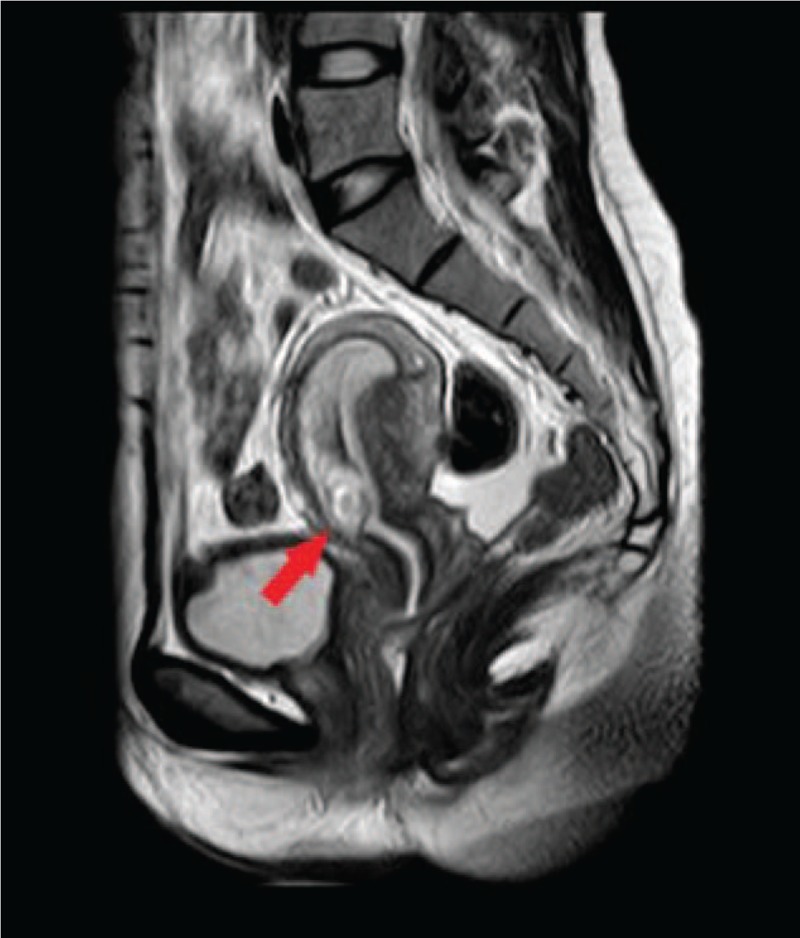
Pelvic magnetic resonance imaging scan showing a group of abnormal cystic components signal on the anterior wall of the uterine isthmus incision.

On March 16, 2016, a 28-year-old woman (gravida 3, para 1) was admitted to our hospital because of amenorrhea for 48 days and vaginal bleeding for 3 days. She had a cesarean section 1 year ago. On admission, she complained of irregular vaginal bleeding and mild abdominal pain. A pelvic examination showed a closed external cervical os and a normal uterus with tenderness. Her hemoglobin and serum human chorionic gonadotropin (hCG) levels were 110 g/L and 7894 IU/L, respectively. A transvaginal sonogram showed a 1.2 × 1.4 × 1.5-cm-sized gestational sac implanted near the previous cesarean scar in the anterior wall of the uterine corpus (Fig. 1). The gestational sac was bulging toward the serosa, with a 5-mm-thin layer of overlying myometrium. A pelvic magnetic resonance imaging (MRI) scan showed a group of abnormal cystic component signal on the anterior wall of the uterine isthmus incision, measuring approximately 1.6 × 2.0 × 1.5 cm (Fig. 2). The lesions, low signal on T1-weighted image (T1WI), and mixed with high signal on T2WI, were prominent in the uterine cavity. The myometrium of the anterior wall of the uterine isthmus incision was not continuous, with the thinnest area at approximately 0.6 cm, whereas the uterine serosa was continuous. Based on these findings, she was suspected of having CSP.

**Figure 3:**
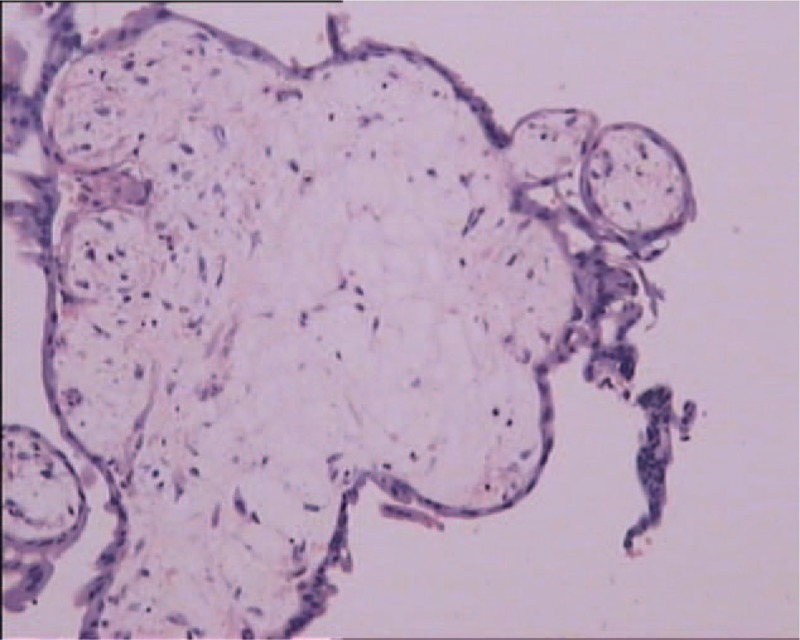
Enlarged hydropic villi and focal trophoblastic proliferation (asterisk) are observed.

On day 1, considering the high risk of hemorrhage, bilateral uterine arterial embolization was performed. Her serum hCG level was 10,940 IU/L. The ultrasound revealed decreased vascularity. On day 2, careful suction evacuation under ultrasound guidance was performed. A 10-g tissue without chronic villi or cystic mole was obtained, which was sent for histologic examination. At the end of the procedure, the ultrasound showed no evidence of the previous lesion. The total blood loss was 5 mL, and oxytocin was intravenously administered to reduce the risk of bleeding. On day 3, vaginal bleeding was not observed, and her serum hCG level decreased to 4488 IU/L, and the patient was discharged. Surprisingly, the histologic examination of the tissue confirmed a partial hydatidiform mole. Chorionic villi with focal trophoblastic proliferation and hydropic change were observed (Fig. 3). The immunohistochemical results showed P57(+) and proliferation index of trophocyte Ki67 of 10%. The patient had a partial molar CSP instead of normal CSP, which we considered preoperatively. The patient's serum hCG level was monitored weekly. Her serum hCG level gradually returned to normal, and she had no vaginal spotting for 9 weeks. Menstruation restarted in the ninth week.

## 3 Discussion

Molar CSP, similar to that observed in our case, is exceedingly rare and may have a high risk of uncontrolled hemorrhage or uterine rupture. However, evidence on the optimal management of molar CSP is lacking, and currently available evidence is based on some case series or individual case reports. Considering its extremely low incidence, we performed a literature search using PubMed with “cesarean scar pregnancy” and “hydatidiform mole” to identify similar reports. Only 3 cases were found,^[3–5]^ and to date, 2 of these cases were partial molar CSP and the other one remained unknown. The 3 reports are listed in Table 1.

**Table 1:**
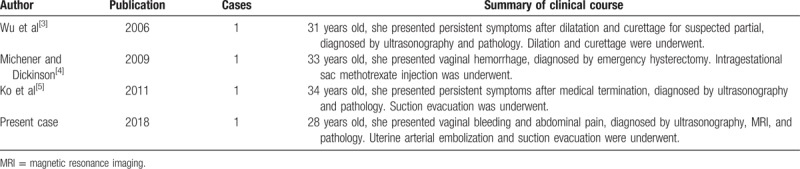
Summary of clinical case reviews of molar pregnancy in the cesarean scar.

Wu et al^[3]^ reported the first molar CSP case in 2006. The patient was treated with dilation and suction curettage. However, the complex mass near the cesarean scar was not found in time, causing a repeat dilation and suction curettage.

Michener and Dickinson^[4]^ reported the second case in 2009, wherein the patient was treated with intragestational sac methotrexate injection. The patient underwent emergency hysterectomy as intervention owing to the recurrence of vaginal bleeding after 10 months.

In 2012, Ko et al^[5]^ reported the third case, wherein transvaginal ultrasonography findings led to the suspicion of molar pregnancy. A complex lesion with cystic spaces and increased vascularity was detected by sonogram. Bilateral femoral arterial sheaths were inserted preoperatively, and ultrasound-guided suction evacuation was performed. Tissue was obtained, and histologic examination confirmed a partial hydatidiform mole. The patient was followed-up for 7 weeks until serum hCG level returned to normal.

Based on previous and our cases, clinical or imaging manifestation of molar CSP includes menopause, vaginal hemorrhage, abdominal pain, increasing serum hCG level, or persistent symptoms after termination, and a gestational sac with cystic spaces detected by ultrasonography. Differentiating molar CSP from normal CSP is difficult because of common symptoms such as amenorrhea and vaginal bleeding.^[6]^ The diagnosis of molar pregnancy in other sites is often made based on typical symptoms and signs, serum hCG levels, and imaging, whereas histologic evidence is not necessary.^[7]^ The most commonly used imaging examinations include those with ultrasound and MRI.

Ultrasound is widely performed in early pregnancy, which may diagnose uterine molar pregnancy. The typical ultrasound feature is a snow storm, besides fetal development seems delayed, and fetus may be malformed in partial hydatidiform mole.^[2]^ However, Fowler et al^[8]^ reported that only 40% to 60% of all molar pregnancies are suspected on ultrasound and after the 14th gestational week. However, the sonogram of all reported and our present cases revealed no snow storm, only a complex mass near the previous cesarean scar. Thus, diagnosis of cesarean scar molar pregnancy by ultrasound may be difficult, particularly for patients in early pregnancy.

In our case, MRI was performed to further evaluate the mass and blood flow near the cesarean scar. The result showed a group of abnormal cystic components signal on the anterior wall of the uterine isthmus incision, which was suspected to be a CSP. Yamada and Ohira^[9]^ reported a preoperative diagnosis of ectopic molar pregnancy. MRI revealed a right cornual mass with isosignal intensity on T1WI and high signal intensity on T2WI, and they detected hydropic villi that are small vesicles in the mass presenting low signal intensities on T1WI and high signal intensities on T2WI. In their opinion, ectopic molar pregnancy is characterized as molar tissue-like tiny cystic lesions, intratumoral hypervascularity, and dense enhancement on MRI. However, this was the only case reported wherein ectopic molar pregnancy was diagnosed before surgery using MRI, and further investigation using MRI in the preoperative diagnosis is needed.

At diagnosis, the serum hCG level is often higher in molar pregnancy than in normal pregnancy, and the serum hCG level in complete hydatidiform mole is significantly higher than that in partial hydatidiform mole. In complete hydatidiform mole, the serum hCG level is often >100,000 IU/L at the average diagnosed gestational ages of 11.9 weeks.^[10]^ In partial hydatidiform mole, the serum hCG is >100,000 IU/L in <10% of cases.^[11]^ The serum hCG levels in previous and our cases ranged from 7894 to 30,756 IU/L, suggesting that molar CSP could not be diagnosed with serum hCG levels alone.

Considering the limitations of ultrasound, MRI, and serum hCG levels in the differential diagnosis of molar CSP from normal CSP, postoperative specimen should be sent for histologic examination and genetic evaluation, if necessary. Once molar pregnancy is confirmed, molar pregnancy should be managed accordingly.

## Author contributions

**Conceptualization:** Xianrong Qi.

**Writing – original draft:** Chen Ling, Jitong Zhao.

**Writing – review & editing:** Chen Ling, Xianrong Qi, Jitong Zhao.
