# Partial molar pregnancy in the cesarean scar: A case report and literature review: Erratum

**DOI:** 10.1097/MD.0000000000012627

**Published:** 2018-09-21

In the article, “Partial molar pregnancy in the cesarean scar: A case report and literature review”,^[1]^ which appeared in Volume 97, Issue 26 of *Medicine*, Dr Xiarong Qi's name appeared incorrectly as Xianrong Qi.
